# Flexible promoter architecture requirements for coactivator recruitment

**DOI:** 10.1186/1471-2199-7-16

**Published:** 2006-04-28

**Authors:** Derek Y Chiang, David A Nix, Ryan K Shultzaberger, Audrey P Gasch, Michael B Eisen

**Affiliations:** 1 Department of Molecular and Cell Biology, University of California, Berkeley, CA 94720, USA; 2 Department of Genome Sciences, Life Sciences Division, Ernest Orlando Lawrence Berkeley National Lab, Berkeley, CA 94720, USA; 3 Department of Genetics, University of Wisconsin, Madison, WI 53706, USA; 4 Broad Institute of MIT and Harvard, Cambridge, MA 02141, USA; 5 Affymetrix, Santa Clara, CA 95051, USA

## Abstract

**Background:**

The spatial organization of transcription factor binding sites in regulatory DNA, and the composition of intersite sequences, influences the assembly of the multiprotein complexes that regulate RNA polymerase recruitment and thereby affects transcription. We have developed a genetic approach to investigate how reporter gene transcription is affected by varying the spacing between transcription factor binding sites. We characterized the components of promoter architecture that govern the yeast transcription factors Cbf1 and Met31/32, which bind independently, but collaboratively recruit the coactivator Met4.

**Results:**

A Cbf1 binding site was required upstream of a Met31/32 binding site for full reporter gene expression. Distance constraints on coactivator recruitment were more flexible than those for cooperatively binding transcription factors. Distances from 18 to 50 bp between binding sites support efficient recruitment of Met4, with only slight modulation by helical phasing. Intriguingly, we found that certain sequences located between the binding sites abolished gene expression.

**Conclusion:**

These results yield insight to the influence of both binding site architecture and local DNA flexibility on gene expression, and can be used to refine computational predictions of gene expression from promoter sequences. In addition, our approach can be applied to survey promoter architecture requirements for arbitrary combinations of transcription factor binding sites.

## Background

In most eukaryotes, the sequences that regulate transcription integrate multiple signals, through the binding of different transcription factors, to modulate levels of gene expression. When bound to DNA, transcription factors anchor the assembly of multiprotein complexes that influence the recruitment of RNA polymerase. Efficient assembly depends on optimally spaced protein-protein interactions among transcription factors and auxiliary proteins [1-4]. Since transcription factors recognize specific sites on DNA, the distance between these binding sites can influence how transcription factors interact with each other and other proteins. For example, overlapping sites may prevent two transcription factors from binding simultaneously, while sites too distant from each other may hinder bound transcription factors from recruiting necessary cofactors. Furthermore, some distantly spaced sites can only properly interact when the DNA between them is looped, a process influenced by the composition of the looped DNA.

Computational approaches take into account the multifactorial nature of transcriptional regulation when discovering transcription factor binding sequence motifs. Several methods search for pairs of enriched sequences, while allowing either fixed or variable spacing between them [5-8]. Other approaches start with binding specificities for pairs of known transcription factors, then assess whether the predicted occurrences of their binding sites are closely spaced [9-11]. Notably, most of these methods assess the spacing between binding sites with arbitrary criteria, such as minimum and maximum distance thresholds between binding sites. However, the biological relevance and suitability of these thresholds has seldom been verified experimentally.

Several mechanisms that govern transcription factor interactions have been previously described. Transcription factors may bind cooperatively to adjacent sites in DNA, thus increasing the stability of the ternary DNA-protein complex. Since this effect is mediated by direct protein-protein interactions, sites for cooperatively binding transcription factors are usually spaced within 20 bp (*e.g*., [12-15]). Slight alterations in spacing between the binding sites can drastically reduce gene expression unless helical phasing is preserved. Computational analyses suggest that proper helical phasing between predicted binding sites may be a common property of transcriptional control regions [16,17].

Alternatively, transcription factors may bind to DNA independently and cooperatively recruit a coactivator protein. Co-recruitment of such activators is analogous to an "AND gate" in logic. Coincident binding of two proteins increases the fidelity and specificity of signal detection [2,18,19]. The network of transcription factors that regulates sulfur gene derepression in yeast provides a model system to dissect the promoter architecture requirements for coactivator recruitment. Among these transcription factors, only the coactivator Met4 contains a strong activation domain. However, Met4 does not bind to DNA directly, but is recruited in sulfur limitation conditions by Cbf1 and Met28 to the *MET16 *promoter, as well as by Met28 and Met31/32 on regions from the *MET3 *and *MET28 *promoters [20,21]. In addition, yeast two-hybrid assays with Met4 truncation mutants revealed distinct regions that mediate interaction with Cbf1 and Met31/32 [21,22]. Taken together, these studies suggest a model by which the coactivator Met4 is coordinately recruited by the transcription factors Cbf1, Met28 and Met31/32 to the promoters of sulfur utilization genes (reviewed in [23]). Nevertheless, the effects of distance constraints and sequence context between Cbf1 and Met31/32 binding sites have not been characterized.

We use the term promoter architecture to refer to distance constraints and sequence context effects that govern interactions among transcription factor binding sites. Our goal is to understand how promoter architectures differ for transcription factors that participate in coactivator recruitment, versus those that bind cooperatively. In this work, we developed a synthetic promoter assay to characterize how various distances between Cbf1 and Met31/32 binding sites influenced gene expression in response to methionine starvation. The relative order of binding sites affected reporter gene expression. We discovered that distance constraints on coactivator recruitment were more flexible than those for cooperatively binding transcription factors. Distances from 18 to 50 bp between binding sites could support efficient recruitment of Met4, with only slight modulation by helical phasing. Intriguingly, we found that certain sequence contexts between the binding sites abolished gene expression. Finally, we noted that the probability of coactivator recruitment could be affected by the bendability of the spacer sequence between transcription factor binding sites.

## Results

### Annotated promoters of sulfur-regulated genes contain closely spaced binding sites

**Figure 1:**
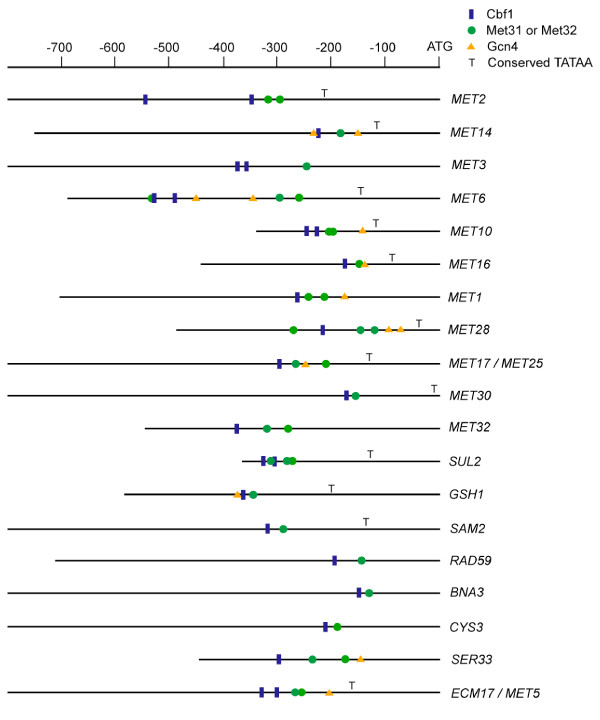
**Promoter architectures of annotated sulfur-regulated genes**. Conserved binding sites for Cbf1 (blue rectangles, TCACGTG), Met31 or Met32 (green circles, TGTGGC), Gcn4 (orangle triangles, TGA [C|G]TCA) and TBP (TATAA) are drawn to scale in the indicated intergenic regions. A binding site was considered conserved if invariant copies were aligned in at least 3 out of 4 closely-related *Saccharomyces *species in a multiple sequence alignment [24, 49].

We first surveyed the promoter architectures of 19 annotated genes in *S. cerevisiae *that were co-expressed under sulfur-limiting conditions [24]. All of these promoters contained Cbf1 and Met31/32 binding sites with perfectly identical sequences among at least 3 of 4 closely-related yeast species (Figure 1). We assessed binding site conservation based on identity within a multiple sequence alignment, but not the distances between the sites. Due to the small sample size, we did not distinguish between the possible relative orientations of the binding sites. Notably, each sulfur-regulated promoter included a Cbf1 binding site upstream of a Met31/32 binding site. These conserved binding sites could occur in either forward or reverse orientation.

**Figure 2:**
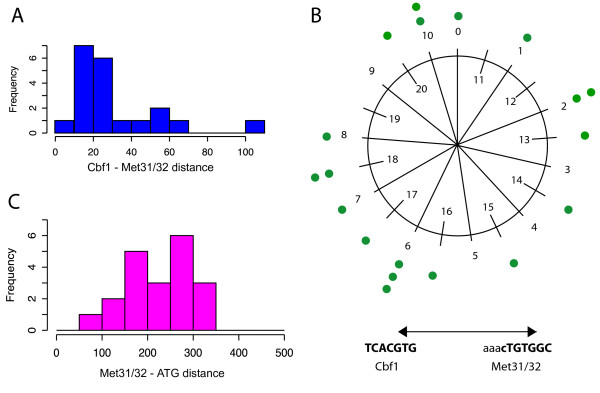
**Minimum distances between conserved Cbf1 and Met31/32 binding sites in annotated sulfur-regulated promoters**. (A) Histogram of minimum distances between a Cbf1 binding site (TCACGTG) and a Met31/32 (TGTGGC) binding site. Distances were calculated from the center of each binding site, as indicated by the arrows between the consensus sequences. (B) Helical wheel projection of minimum distances. Cbf1 binding sites were aligned at the top of the helical wheel (position 0). Each green dot represents the remainder of a minimum distance from (A) divided by 10.5 bp. Since the helical pitch of DNA is 10.4 bp, each dot approximates the position of the Met31/32 binding site relative to the Cbf1 binding site. (C) Histogram of distances between the Met31/32 binding sites from (A) and the translation start site.

In each promoter, we searched for the Cbf1 binding site upstream of the Met31/32 binding site that yielded the smallest distance between them in *S. cerevisiae*. A histogram of these smallest distances showed a peak between 10 and 30 bp (Figure 2A). This peak suggested an optimal distance between the transcription factors was necessary for efficient Met4 recruitment. When investigating whether the distances between the closest pairs of binding site were helically phased, we could not detect an enrichment of distances on a certain face of DNA (Figure 2B). Finally, the vast majority of annotated promoters contained Met31/32 binding sites within 100 to 350 bp upstream of the translation start site (Figure 2C).

### Cbf1 binding sites are required upstream of Met31/32 binding sites

**Figure 3:**
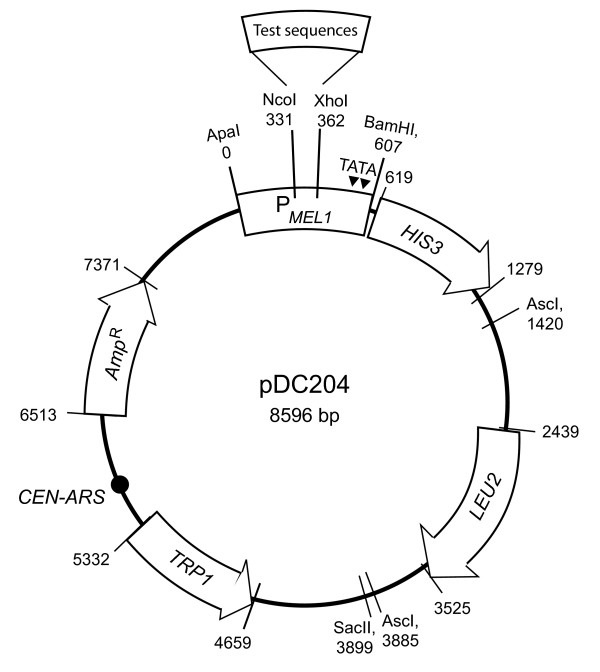
**Synthetic promoter system**. A minimal promoter from the *MEL1 *gene (*P*_*MEL1*_) was fused upstream of a *HIS3 *reporter gene on a single-copy plasmid. Selected restriction enzyme sites are labeled with their coordinates. Various combinations of Cbf1 and Met31/32 binding sites were inserted between the NcoI and XhoI restriction enzymes sites in the minimal promoter.

A larger collection of sulfur-regulated promoters would provide more statistical power to define key components of promoter architecture. To explore sequence space more diverse than that found in the yeast genome, we developed a synthetic genetic approach to select for sulfur-regulated promoters from a plasmid library. We engineered a single-copy plasmid that fused a minimal promoter upstream of the *HIS3 *reporter gene (Figure 3). To test their effects on transcription levels, different promoter architectures were embedded in the context of the minimal promoter from the *S. cerevisiae MEL1 *gene [25]. This promoter was chosen for its low background expression, compared to promoters derived from the *S. cerevisiae CYC1 *gene. Promoter architectures with combinations of regulatory sequences that supported sufficient expression of the *HIS3 *reporter gene enabled the parental yeast strain BY4742 to grow in media lacking histidine. In addition, semiquantitative measurements of *HIS3 *expression can be assayed by titration with 3-amino-1,2,4-triazole (3-AT), a competitive inhibitor of the *HIS3 *gene product [26]. Faster growth rates in the presence of higher concentrations of 3-AT correspond to higher expression levels of the *HIS3 *gene.

**Figure 4:**
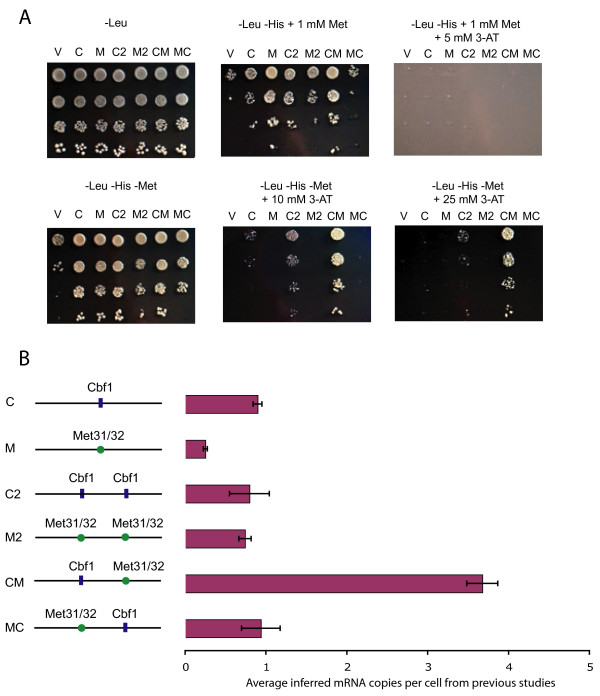
**Reporter gene expression driven by various combinations of Cbf1 and Met31/32 binding sites**. (A) Yeast strains were grown under either repressive (+Met) or activating (-Met) growth conditions, in addition to varying concentrations of the inhibitor, 3-AT. Each column represents a 10-fold serial dilution of a yeast strain containing a reporter plasmid with a different binding site combination, labeled as follows. V: vector alone, C: Cbf1 binding site, M: Met31/32 binding site, C2: Two Cbf1 binding sites spaced by 35 bp, M2: Two Met31/32 binding sites spaced by 35 bp, CM: Cbf1 binding site placed 35 bp upstream of a Met31/32 binding site, MC: Met31/32 binding site placed 35 bp upstream of a Cbf1 binding site. Yeast strains were grown on the indicated media for 5 days at 30°C. Serial dilutions were performed in triplicate, and a representative dilution is displayed for each growth condition. (B) Average inferred transcript levels in sulfur-limitation conditions associated with various promoter architectures. For each gene, the gene expression log ratio between sulfur limitation and complete media conditions was calculated as the average log base 2 expression ratio from previously published gene expression studies [28, 29]. To infer the average number of mRNA transcripts per cell, the gene expression ratio for each gene was multiplied by the basal transcript level as measured during growth in rich media [29]. Inferred transcript levels were averaged over sets of genes that shared the indicated binding site combinations in the 500 bp upstream of their translation start sites; the first row indicates the average for all genes. Error bars indicate the standard error of the mean.

We sought to define the minimal regulatory information that was sufficient to induce reporter gene expression in response to sulfur limitation. As a negative control, none of the synthetic promoters were able to induce enough *HIS3 *expression in a repressive concentration of methionine to support growth on a low level (5 mM) of 3-AT (Figure 4A). In addition, neither the minimal promoter alone nor a single Met31/32 binding site could support growth in the absence of methionine with 10 mM 3-AT. A single Cbf1 binding site supported weak growth on 10 mM 3-AT. In the wild-type *MET14 *promoter, a Cbf1 binding site was found 35 bp upstream from a Met31/32 binding site, as measured by center-to-center distance. Two Cbf1 binding sites placed at the same distance showed moderate *HIS3 *expression. However, two Met31/32 binding sites were unable to support growth. A promoter with a Cbf1 binding site upstream of a Met31/32 binding site showed the highest level of *HIS3 *expression. In contrast, a promoter with the Met31/32 binding site found upstream of the Cbf1 binding site was unable to support growth on 10 mM 3-AT.

We compared these results obtained from a minimal promoter system to the average effects of promoter architecture in endogenous yeast genes. For each gene, we estimated the average number of mRNA copies per cell by multiplying the basal transcript levels in rich growth media and the average ratio of gene expression change from published microarray studies of sulfur limitation conditions [27-29]. We then averaged these inferred absolute transcript levels for sets of genes whose promoters shared combinations of Cbf1 or Met31/32 binding sites (Figure 4B). The presence of Cbf1 binding sites or Met31/32 binding sites was associated with a significant increase in inferred transcript levels compared to the rest of the genome. However, two Cbf1 or two Met31/32 binding sites did not contribute to higher transcript levels. Genes whose promoters shared a Cbf1 binding site upstream of a Met31/32 binding site showed significantly higher transcript levels compared to genes with only a single Cbf1 binding site. In contrast, the opposite order of binding sites was not associated with a significant increase in transcript levels. Thus, the constraint on the order of binding sites was consistent with computational predictions of the regulatory effects for various promoter architectures.

### High cooperativity between Cbf1 and Met31/32 binding sites spaced at least 18 bp apart

We predicted that efficient recruitment of Met4 to the promoters of sulfur utilization genes should depend on the spacing between Cbf1 and Met31/32 binding sites. To investigate the effect of varied spacing on reporter gene activation, we constructed a set of promoter libraries that differed by 2-bp increments from 6 bp to 34 bp, as well as 5-bp increments from 40 bp to 50 bp. Each promoter library had a fixed size but degenerate nucleotide sequences between the Cbf1 and Met31/32 binding sites. The binding sites were flanked by 10 bp of sequence from the *MET16 *promoter of *S. bayanus*, which lacks an adjacent Gcn4 site.

By pooling hundreds of yeast transformants for each library, we reasoned that the contribution of nucleotide composition on Met4 recruitment and subsequent gene activation would be averaged out. Growth rates for each promoter library thus represent the aggregate effect of a certain distance on reporter gene expression. At several steps in the procedure, we took care to reduce the potential of selecting only the fastest-growing strains from each pool. First, we picked transformants of similar colony size. Second, we amplified these pools overnight in dropout leucine media, which ensured that the plasmids were retained but did not select for levels of promoter expression. Third, we measured growth rates for 7 hours after promoter induction and selection with 3-AT, which corresponded to fewer than 2.5 doubling times. Finally, we verified the complexity of the library pools by isolating single colonies on non-selective growth plates and sequencing of the promoters in twenty different colonies.

**Figure 5:**
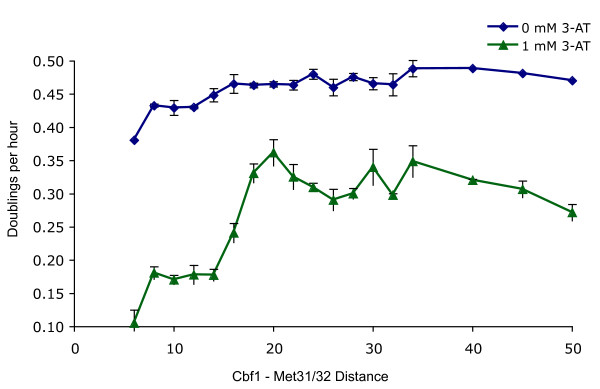
**Average growth rates for sequence libraries with defined distances between Cbf1 and Met31/32 binding sites**. We generated libraries of pooled transformants containing single-copy plasmids with the indicated distances between Cbf1 and Met31/32 binding sites. We induced reporter gene expression by transferring these pooled cultures into media lacking histidine, leucine and methionine, plus the indicated concentration of 3-AT (see Methods for details). The growth rate, expressed in doublings per hour, for each pooled culture was obtained from regular measurements of OD_600_. The average growth rate and standard error of the mean are plotted for three independent trials.

Pooled measurements of growth rates in sulfur limiting conditions determined that a minimum distance between Cbf1 and Met31/32 binding sites was required for the highest levels of gene expression (Figure 5). Yeast harboring promoter libraries of varying sizes grew at similar rates in the absence of 3-AT, indicating low levels of leaky transcription from the reporter construct. Expression levels of the *HIS3 *reporter gene were titrated with the addition of 1 mM 3-AT; similar results were obtained with different concentrations of 3-AT (data not shown). Binding sites whose centers were spaced fewer than 14 bp apart promoted weak reporter gene expression. At these close distances, Cbf1 and Met31/32 may be sterically constrained from assembling a complex with Met4. Reporter gene expression increases sharply as the distance between binding sites is increased from 14 bp to 18 bp. The highest levels of gene expression were observed for promoter libraries with binding sites spaced from 18 bp to 50 bp apart, whereas helical phasing modulated the average growth rate by less than 20%.

### Sequence context between binding sites can inhibit gene activation

**Figure 6:**
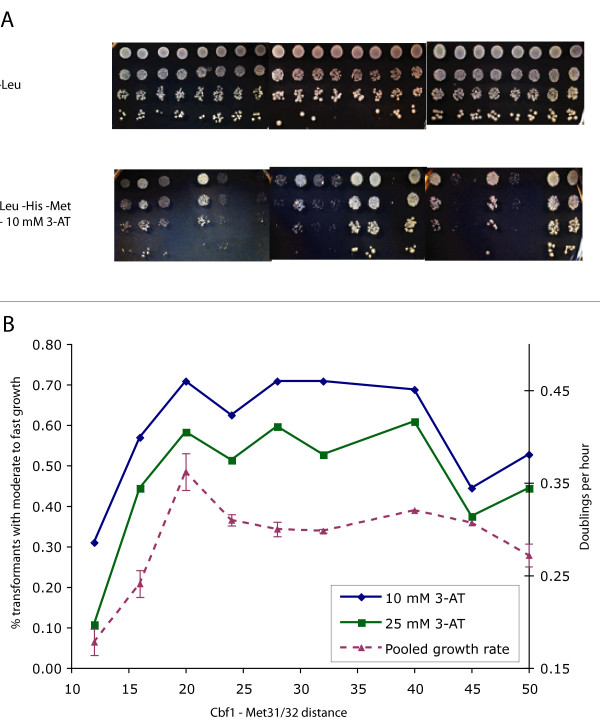
**Different sequences between Cbf1 and Met31/32 binding sites show a range of reporter gene activation**. (A) Serial dilutions of yeast containing reporter plasmids with the same distance (20 bp) between binding sites, but different spacer sequences. Yeast strains were grown on the indicated media for 5 days at 30°C. (B) Proportions of transformants that displayed moderate to high levels of growth on solid media with 10 mM or 25 mM 3-AT. For each distance between binding sites, growth rates of 72 transformants with different spacer sequences were assayed with serial dilutions. Average growth rates in liquid media with 1 mM 3-AT, as in Figure 4, is also shown for comparison purposes.

In addition to characterizing the aggregate effects of binding site spacing, we also examined the effects of different spacer sequences on reporter gene expression. We assayed the growth rates of individual yeast transformants on solid media containing 10 mM or 25 mM 3-AT. Each of the 70 to 72 transformants tested for a certain distance harbored a promoter with a different, random sequence between the Cbf1 and Met31/32 binding sites. We observed reproducible variability in growth rates among transformants with the same distance, but different spacer sequences, between Cbf1 and Met31/32 binding sites (Figure 6).

At each distance surveyed, a certain proportion of intervening sequences was compatible with reporter gene expression. Since the pooled growth rates in liquid media were qualitatively similar over this distance range, we interpret these proportions as the probability that a random intervening sequence would support gene expression at a given distance. At a distance of 12 bp between sites, less than 30% of the sequences supported reporter gene expression. At distances between 16 and 50 bp, the proportion of transformants that showed moderate to high levels of growth on 25 mM 3-AT varied from 38% to 60%. We observed a modest dependence of this proportion on helical phasing in the distance between binding sites.

To investigate what features of spacer sequences correlated with gene activation, we sequenced a sample of 28 promoters with distances of 12 bp, as well as 41 promoters with distances of 20 bp, between the Cbf1 and Met31/32 binding sites (Table 2). Promoters that supported gene expression (positives) were similar in nucleotide composition to promoters that inhibited gene expression (negatives). Since no trimers or tetramers were enriched in the positive or negative promoter sets, additional sequence-specific transcription factors probably did not contribute to gene expression. The most discriminating feature of negative promoters was a shared G or T immediately 5' to the Met31/32 binding site in 15 of 17 examples of distance 12, as well as in all 13 examples of distance 20. However, about half of the positive examples contained a G or T at that position, as expected.

**Figure 7:**
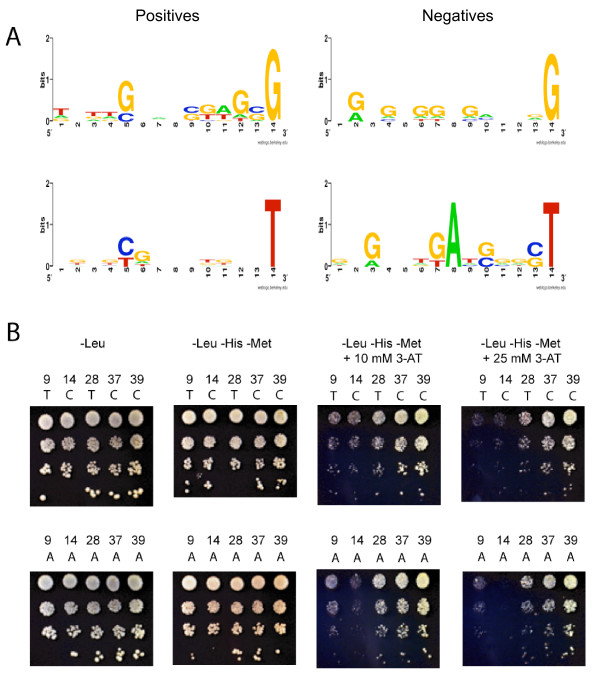
**Sequence context effects were not determined by individual nucleotides**. (A) Sequence logos for a sample of promoters with a center-to-center distance of 20 bp between Cbf1 and Met31/32 binding sites. At each position, the height of the nucleotide corresponds to the information content at that position, which weights its frequency in the sequenced sample compared with its expected frequency [31]. The sample was divided into sequences that supported or inhibited gene activation, as well as whether a guanine or thymine was found adjacent to the Met31/32 binding site. A separate logo was generated by WebLogo for each sub-sample [30]. Since three invariant nucleotides on both the 5' and 3' ends of the spacer sequence represented the Cbf1 and Met31/32 binding sites, respectively, only the central 14 bp are displayed. (B) Mutation of single nucleotides in positive promoters failed to inhibit reporter gene activation *in vivo*. Each column corresponds to a yeast strain containing a different spacer sequence with a distance of 20 bp between the binding sites. The clone number and nucleotide at position 11 are indicated above each panel. Ten-fold serial dilutions were performed in triplicate, and a representative dilution is shown.

We searched for additional residues that could discriminate among sequences that shared a G or T at the most 3' position of the spacer region using WebLogo [30,31]. We compared sequence logos between the positive and negative promoters to calculate whether any nucleotides were enriched at particular positions in the spacer sequences (Figure 7A). By focusing on the three most informative positions, we derived nucleotide combinations that predicted negative promoters with an overall sensitivity of 80% and a specificity of 89% (Table 3).

To test whether the A_11_-T_17 _nucleotide combination was sufficient to inhibit gene expression in spacer sequences of length 20, we identified five promoters with a B_11_-T_17 _sequence combination and converted the nucleotide at position 11 to an adenine by site-directed mutagenesis. Similar levels of reporter gene expression were driven by the original and mutant promoters, as assayed by serial dilutions on media containing 10 mM or 25 mM 3-AT (Figure 7B). Thus, the effects of sequence context are not encoded by specific positions within the primary nucleotide sequence.

## Discussion

### Promoter architecture features of yeast sulfur utilization genes

We have developed a synthetic promoter assay to test how various features of promoter architecture affected *HIS3 *reporter gene expression in the context of a common minimal promoter. Although this reporter gene assay is indirect, it has been successfully used to obtain semi-quantitative measurements of transcript levels [26]. We applied this system to characterize the collaborative recruitment of the coactivator Met4 by the transcription factors Cbf1 and Met31/32 in response to methionine starvation. We found that the relative order of binding sites was crucial, since a Cbf1 binding site was required upstream of a Met31/32 binding site for full gene expression. The influence of Cbf1 and Met31/32 binding site order on reporter gene expression implies that the spatial orientation of the Met4 activation domain is required for the recruitment of downstream targets. Two Cbf1 binding sites could moderately increase reporter gene expression, yet the mechanism for this enhanced activation is unclear. Synergistic activation of reporter gene expression occurred when Cbf1 and Met31/32 binding sites were spaced at least 18 bp apart. Notably, the allowed distances for coactivator recruitment extend beyond the maximal range for cooperatively binding transcription factors. Finally, we discovered that different sequence contexts between binding sites produced considerable heterogeneity of reporter gene expression, whereas helical phasing showed comparatively little effect.

Although the transcription factors Cbf1 and Met31/32 lack canonical activation domains, they can serve as activators via collaborative recruitment of the coactivator Met4 when they are jointly bound to the promoters of sulfur utilization genes (reviewed in [23]). Our genome-wide computational survey found that genes with single Cbf1 or Met31/32 binding sites in their promoters were associated with significantly higher transcript levels, on average, when compared to the rest of the genome (Figure 4B). However, the presence of two binding sites for the same transcription factor was not associated with a further increase in transcript levels on average. In contrast, we found that two Cbf1 binding sites separated by 35 bp in a minimal promoter conferred increased reporter gene expression (Figure 4A). This discrepancy could be explained by distinct distance constraints between two binding sites for the same transcription factor.

Our data could not rule out the possibility that Met31/32 may serve as transcriptional repressors when bound to other promoters that lack Cbf1 binding sites. We found that reporter gene expression in methionine starvation was lower for minimal promoters with one or two Met31/32 binding sites, compared to a minimal promoter with a Cbf1 binding site alone (Figure 4A). Several models could explain how the binding of Cbf1 could convert Met31/32 from a repressor to an activator. Since the recruitment of the coactivator Met4 requires interactions with both Cbf1 and Met31/32, Met4 could displace a corepressor that may be constitutively bound to Met31/32. In contrast, the binding of Cbf1 could recruit enzymes that confer posttranslational modifications on Met31/32. These modifications could induce a conformational change that relieves repressive activity of Met31/32. Kinetic analyses of transcription factor binding and subsequent recruitment of multiprotein regulatory complexes by chromatin immunoprecipitation could help distinguish between these models.

### Distinct promoter architecture requirements for different transcription factor combinations

The promoter architecture requirements for Met4 coactivator recruitment differ considerably from previously characterized yeast promoters. For instance, the transcription factor Rap1 can efficiently recruit Gcr1/2 only when their binding sites are found 13 or 23 bp apart [13]. Notably, a distance of 18 bp that altered the helical phasing between these factors abolished gene activation. Similarly, helical phasing between Pho2 and Swi5 binding sites modulates cooperative binding by almost three-fold [14]. As an extreme case, the insertion of a single base pair between the a1 and α2 binding sites abolishes cooperative binding [15]. In contrast with the above transcription factor pairs that bind cooperatively, levels of reporter gene activation were fairly consistent when Cbf1 and Met31/32 binding sites were spaced between 18 bp and 50 bp apart. The tolerance of Met4 coactivator recruitment on a wide distance range contradicts the model that transcription factor interactions are predominantly determined by the precise spacing between their binding sites. Intriguingly, the recruitment of Met4 to a common minimal promoter seems to depend more on the sequence context between Cbf1 and Met31/32 binding sites than on the distance between them, provided that the minimum distance requirements were met. In light of these results, previous studies that varied distances between transcription factor binding sites should be reassessed, since they usually considered only a single sequence context for each distance.

The rather flexible distance constraints between Cbf1 and Met31/32 binding sites suggest that Met4 recruitment may not require rigid, simultaneous protein-protein interactions among the bound transcription factors. Taken together, these experiments suggest that the process of Met4 recruitment differs considerably from the lock-and-key arrangements of bound transcription factors that govern the mammalian interferon beta enhanceosome [1,2]. Instead, an intrinsic property of the intervening sequence context, such as DNA bendability, may facilitate an induced fit between the bound transcription factors and Met4. Whereas the distance between binding sites plays a diminished role in bridging bound transcription factors, intervening sequences with low intrinsic bendability could impair coactivator recruitment. Thus, the key requirements of promoter architecture may rely heavily on the molecular mechanism of transcription factor interactions at a particular set of co-regulated promoters.

### Possible effects of sequence context between transcription factor binding sites

Sequence context could alter Met4 recruitment in several ways. First, residues adjacent to binding sites could reduce the binding affinity of Cbf1 or Met31/32. Accordingly, we found that all spacer sequences that were incompatible with reporter gene expression contained a guanine or thymine immediately 5' to the Met31/32 binding site. Secondly, the DNA bendability of the spacer sequence could alter the conformation of Cbf1, which bends DNA by approximately 68° [32]. Conformational changes in Cbf1 could affect its protein-protein interactions with Met28 or Met4, thus reducing Met4 recruitment. A requirement for DNA bendability on protein-protein interactions has been recently shown for the transcription factor Mcm1, which bends DNA by 66°, comparable to the bend angle induced by Cbf1 [33]. A point mutant of Mcm1 with a DNA bending angle of 46° had a lower affinity for cooperative binding with Fkh2 than a mutant with a DNA bending angle of 49°, suggesting that a certain threshold of DNA bending was required for ternary complex formation *in vitro *[33]. Circular permutation assays on promoters with different sequence contexts could test whether the extent of bendability correlates with reporter gene activation. In addition, chromatin immunoprecipitation studies could identify the transcription factors whose binding *in vivo *is affected by sequence context.

Whereas the influence of sequence context on gene activation has been widely reported *e.g*., [34-36], the key determinants of sequence context have been poorly defined. Except for the residue adjacent to the Met31/32 binding site, we could not identify features of the primary nucleotide sequence that correlated with gene activation. Previous studies have reported that protein-DNA interactions can be affected by physicochemical properties of DNA, such as twist [37]. Although we assessed several dinucleotide parameters, we could not find any significant correlation between the average parameter value of a spacer sequence and reporter gene activation (data not shown).

Epigenetic effects could account for some of the observed variability in gene activation among promoters with different sequence contexts. By examining multiple independent serial dilutions for several promoter sequences (Supplementary Figure 1), we believe that this variability is reproducible and not due to stochastic effects on individual clones. In order to sample a large number of promoter architectures, we assayed reporter gene expression from a single-copy plasmid, which yields over 10,000-fold higher transformation efficiency than chromosomal integration. We have not explored how the flanking sequence composition of wild-type promoters may affect the basal or Met4-induced nucleosomal accessibility of Cbf1 and Met31/32 binding sites in the genome. Cbf1 can also modulate nucleosome positioning and recruit the Isw1 chromatin remodeling complex [38,39]. Thus, additional determinants of local sequence context that affect the binding or DNA bending of Cbf1 may influence Met4 recruitment and gene activation in a chromosomal context.

### Implications for computational predictions of transcription factor interactions

The development of computational methods to predict the transcriptional output of an arbitrary regulatory sequence has attracted considerable interest, as reviewed in [40,41]. Most computational approaches assess the enrichment of predicted binding sites within a large sequence region, while ignoring the spatial arrangement of the binding sites. Moreover, only a handful of methods explicitly consider whether binding sites are more closely spaced than expected [9,10,42-44]. These methods typically specify minimum and maximum distance thresholds between which transcription factors are predicted to interact. Whereas the use of thresholds roughly approximates the range of transcription factor interactions, our above experiments suggest two major improvements for more accurate predictions.

First, different mechanisms of transcription factor interactions may impose distinct distance constraints between their binding sites. We found that the minimum spacing between Cbf1 and Met31 binding sites was the key distance constraint on reporter gene activation. We interpret this minimum distance to be a consequence of the coactivator's role in bridging the bound transcription factors. Various coactivators likely have different minimum distance requirements, based on their size and the relative locations of their interaction surfaces with DNA-bound transcription factors. Experimental studies on the promoter architecture requirements for other common coactivators should provide empirical distance thresholds that could improve the prediction accuracy of their regulated target genes.

In addition, the pronounced effects of sequence context on reporter gene activation suggest that highly accurate predictions of target gene regulation may not be easily extrapolated from targeted experimental studies. Further investigations of promoter architecture may benefit from a framework that formalizes how enthalpy gains from protein-protein interactions are offset by the entropy loss of multiprotein complex formation. Thermodynamic measurements on promoter variants with different spacing and sequence contexts between transcription factor binding sites could then be associated with changes in gene activation. Such a theory on the energetics of multiprotein complex formation could provide the quantitative precision needed to predict how a particular transcriptional control region adopts a conformation that enables transcriptional activation.

## Conclusion

The main goal of this work was to characterize the influence of various components of promoter architecture on transcription factor interactions. We found that the requirements for Met4 coactivator recruitment were considerably more flexible than those for cooperatively binding transcription factors. The characteristic requirements of Met4 recruitment included the precise order of Cbf1 and Met31/32 binding sites, a large distance range between the binding sites that was insensitive to helical phasing, and the pronounced inhibitory effects of sequence context. Given the modular design of our synthetic promoter system, our approach can be readily used to characterize the promoter architecture constraints between arbitrary combinations of yeast transcription factors.

## Methods

### Plasmid construction

Plasmid pDC204 was constructed in five steps. 1) The *HIS3 *coding region was PCR amplified from *S. cerevisiae *genomic DNA using the primers HIS3_F_BamHI and HIS3_R (Table 1) and cloned downstream of the *MEL1 *minimal promoter (*P*_*MEL1*_) by ligating into the BamHI + EcoRV-cleaved plasmid YIpMELβ2 from EUROSCARF [25]. Two changes were then made to the *MEL1 *minimal promoter. 2) An NcoI site was introduced into *P*_*MEL1 *_31 bp upstream of the existing XhoI site by site-directed mutagenesis (oligos MEL1_NcoI_W and MEL1_NcoI_C). 3) An out-of-frame ATG codon located 17 bp upstream of the *HIS3 *coding region was removed by site-directed mutagenesis (oligos ATG_W and ATG_C). 4) The *P*_*MEL1*_*-HIS3 *fusion construct was PCR amplified (primers pMH14-F_ApaI & pMH14-R_AscI-SacII) and cloned into the ApaI + SacII-cleaved plasmid pRS314 [45]. 5) The *Kluyveromyces lactis LEU2 *gene was PCR amplified from pUG73 (primers pUG73_F and pUG73_R) [46] and cloned into the AscI site of the above plasmid. Restriction digests confirmed the same-strand orientation of the HIS3 and LEU2 coding regions, and sequencing verified the promoter and coding regions.

### Promoter library construction

Degenerate oligonucleotides were designed with a Cbf1 binding site at a fixed distance upstream of a Met31/32 binding site (Operon) (Table 1). Ten bp of flanking sequence upstream of the Cbf1 binding site and downstream of the Met31/32 binding site were included from the wild-type *MET16 *promoter. Double-stranded DNA was synthesized by Bio-X-Act polymerase (Bioline) from the primer MET16_reverse (Table 1), digested with NcoI and XhoI and ligated into pDC204.

### Yeast strains and media

Strain BY4742 (*MATα his3Δ1 leu2Δ0 lys2Δ0 ura3Δ0*) was obtained from Invitrogen. Growth media were prepared by mixing yeast nitrogen base (Bio101), 2% dextrose and amino acid supplements lacking leucine or lacking histidine, leucine and methionine (BD Biosciences). Histidine or 3-amino-1,2,4-triazole (3-AT) (Sigma) were supplemented to the indicated concentrations.

### Pooled growth rates for promoter libraries

Plasmids containing promoter libraries with the indicated spacings between binding sites were introduced into the BY4742 parental strain by lithium acetate transformation [47]. Transformants that harbored these single-copy plasmids were selected by growth on dropout medium lacking leucine. For each growth rate experiment, over 100 yeast colonies from a separate transformation were pooled and amplified by culturing overnight in dropout media lacking leucine.

To induce reporter gene expression, the pooled yeast cultures were diluted to early log phase (OD_600 _~ 0.04) in 20 mL of dropout media lacking leucine, histidine and methionine and grown at 30°C with shaking at 250 rpm for 3 hours (OD_600 _~ 0.1). Each culture was then split in half and 3-AT was added to one half, to a final concentration of 1 mM. To acclimatize the yeast cultures to 3-AT, the cultures were grown for a further 2.5 hours. Subsequently, we measured the OD_600 _of each pooled culture every 45 minutes until 7 hours after 3-AT addition. These measurements were transformed to log (base 2) values and a linear regression was calculated in Excel. The doublings per hour corresponds to the slope of the linear regression for a single growth curve.

### Computational association of promoter architectures with gene expression

The regulatory information associated with a particular sequence – such as a transcription factor binding site – can be quantified as the average change in gene expression for all genes that contain that sequence in its transcriptional control region. We followed the standard practice of defining yeast transcriptional control regions as the 500 bp upstream of each coding region, as obtained from the Saccharomyces Genome Database [48]. We searched for exact matches to the core recognition sequences for Cbf1 (TCACGTG) or Met31/32 (TGTGGC) on either strand of these upstream regions.

We first computed the relative ratio of transcript levels between sulfur starvation and complete media for each gene. The relative ratio for each gene was obtained by averaging the log base 2 expression ratios for the first four timepoints of an amino acid starvation microarray experiment, as well as four replicates of a 1 mM cadmium treatment [28,29]. To convert these average relative ratios to absolute mRNA levels, we multiplied these relative ratios by the average basal transcript levels in rich growth media as reported by [27]. We reported the average of these mRNA levels for all genes that shared each promoter architecture under consideration.

## Authors' contributions

DYC, DAN, APG and MBE conceived and designed the experiments; DYC performed the experiments; DYC, DAN and RKS analyzed the data; DYC, DAN, RKS, APG and MBE wrote and approved the manuscript.

## Supplementary Material

Additional File 1 **Reproducibility of growth assays**. Two different sequence contexts were tested between Cbf1 and Met31/32 binding sites with a center-to-center distance of 20 bp. The sequences of these clones were confirmed by isolating plasmids and sequencing. Clones 8A and 8B had the sequence TCACGTGTTTACAAACTAGGGGCCACA; clones 12A and 12B had the sequence TCACGTGGGCATTTATGGGAAGCCACA. These plasmids were transformed independently into yeast strains. Serial dilutions of separate isolates were plated on the indicated growth media. Click here for file

## Figures and Tables

**Table 1:** List of oligonucleotides used in this study

Plasmid construction	
Oligo name	Sequence (5'→ 3') [restriction sites underlined]

HIS3_F_BamHI	CGGGATCCCGAAGATGACAGAGCAGAAAGC
HIS3_R	CCTCGTTCAGAATGACACG
MEL1_NcoI_W	CCCTGAAAGGTTTTTCCATGGAATAGTCAGGACGC
MEL1_NcoI_C	GCGTCCTGACTATTCCATGGAAAAACCTTTCAGGG
ATG_W	GTAATAAAAGCAACGACGTTGAACGGATCCCGAAAG
ATG_C	CTTTCGGGATCCGTTCAACGTCGTTGCTTTTATTAC
pMH14-F_ApaI	ATAGGGCCCGGAAATTTGTGTAAAACCCCC
pMH14-R_AscI-SacII	AACAACCGCGGATAATGGCGCGCCCCTCGTTCAGAATGACACG
pUG73_F	AAGGCGCGCCGCATAGGCCACTAGTGGATCTG
pUG73_R	AGTAAGGCGCGCCCAGCTGAAGCTTCGTACGC

	
Promoter library construction	

Oligo name	Sequence (5'→ 3') [restriction sites underlined]

MET16_reverse	CCGCTCGAGTTACTGAAGTTG
Cbf1_6_Met31	CATGCCATGGTATCATCATTTCACGTGGCCACAACTTCAGTAACTCGAGCGG
Cbf1_8_Met31	CATGCCATGGTATCATCATTTCACGTGGNNCCACAACTTCAGTAACTCGAGCGG
Cbf1_10_Met31	CATGCCATGGTATCATCATTTCACGTGGNNNNCCACAACTTCAGTAACTCGAGCGG
Cbf1_12_Met31	CATGCCATGGTATCATCATTTCACGTGGNNNNNNCCACAACTTCAGTAACTCGAGCGG
Cbf1_14_Met31	CATGCCATGGTATCATCATTTCACGTGGNNNNNNNNCCACAACTTCAGTAACTCGAGCGG
Cbf1_16_Met31	CATGCCATGGTATCATCATTTCACGTGGNNNNNNNNNNCCACAACTTCAGTAACTCGAGCGG
Cbf1_18_Met31	CATGCCATGGTATCATCATTTCACGTGGNNNNNNNNNNNNCCACAACTTCAGTAACTCGAGCGG
Cbf1_20_Met31	CATGCCATGGTATCATCATTTCACGTGGNNNNNNNNNNNNNNCCACAACTTCAGTAACTCGAGCGG
Cbf1_22_Met31	CATGCCATGGTATCATCATTTCACGTGGNNNNNNNNNNNNNNNNCCACAACTTCAGTAACTCGAGCGG
Cbf1_24_Met31	CATGCCATGGTATCATCATTTCACGTGGNNNNNNNNNNNNNNNNNNCCACAACTTCAGTAACTCGAGCGG
Cbf1_26_Met31	CATGCCATGGTATCATCATTTCACGTGGNNNNNNNNNNNNNNNNNNNNCCACAACTTCAGTAACTCGAGCGG
Cbf1_28_Met31	CATGCCATGGTATCATCATTTCACGTGGNNNNNNNNNNNNNNNNNNNNNNCCACAACTTCAGTAACTCGAGCGG
Cbf1_30_Met31	CATGCCATGGTATCATCATTTCACGTGGNNNNNNNNNNNNNNNNNNNNNNNNCCACAACTTCAGTAACTCGAGCGG
Cbf1_32_Met31	CATGCCATGGTATCATCATTTCACGTGGNNNNNNNNNNNNNNNNNNNNNNNNNNCCACAACTTCAGTAACTCGAGCGG
Cbf1_34_Met31	CATGCCATGGTATCATCATTTCACGTGGNNNNNNNNNNNNNNNNNNNNNNNNNNNNCCACAACTTCAGTAACTCGAGCGG

**Table 2:** Promoter sequences associated with reporter gene activation

Clone	Growth rate	Intervening sequence
5	-	TGGGTT
8	-	GAGGCG
20	-	GAGCAT
22	-	TGGATG
30	-	GTGAGT
32	-	AAAGAG
33	-	GTGACT
35	-	TGGTGT
36	-	AGAATG
44	-	TGCACT
47	-	AATTGG
48	-	AAACTC
3	+	TAAGAG
6	+	CATAGT
19	+	CGGTCC
25	+	GTTAAT
42	+	CGTCGT
2	++	CGCGTT
4	++	AACCGC
7	++	TGAGGC
27	++	CAAACG
43	++	CCATGG
46	++	GGTTGT
18	+++	ATTGGC
23	+++	AGGCAA
29	+++	ATATAT
31	+++	AAATGA
34	+++	TTGTGA

		

Clone	Growth rate	Intervening sequence

2	-	GAGTCTGATGGTCT
7	-	TGGGTTGTCAACGG
23	-	GGGCAATCGCGATG
28	-	CGTGGGGTGCTTAG
31	-	TATAAGGCGTTGGG
47	-	CGAGGGGAAAACAG
48	-	TGAGGAGATGAAGT
68	-	GAAGTGAGGAGCGG
69	-	AAGAATTACCCGGT
3	+	CCTGATGCCTACAG
16	+	CAAGGCTAGGAGCG
24	+	GCGCAGGATCGGCT
67	+	GGGTGTGAAGGGCT
9	++	TGAGCTCTTGACAT
14	++	GGTTCAACGTTACT
30	++	GCAAGGAGCGAGGG
32	++	AGGGGAACGGAGAG
33	++	TAGTGGGATTTGCG
34	++	GGTGACTAGGCCTC
46	++	GAAGTGGATTGCGT
5	+++	GGACGTAATTTCAA
8	+++	TTTACAAACTAGGG
10	+++	CGATGTACTGCCAA
11	+++	GTTTGTTGGGATGG
12	+++	GGCATTTATGGGAA
13	+++	CCCTTCCTGTGGGC
15	+++	GGTGGTTCATGGGA
18	+++	CGCGCGGGCGTCTT
25	+++	TCAGGGTTCAGCCA
26	+++	CGCGCCGAACGGGC
27	+++	TAGTGTCGGGGGCG
29	+++	GTGGTAGACGCTGC
35	+++	TTATGGTACCACCA
36	+++	TCATGCGTCGTACG
37	+++	TTGCTGGCAAGGAT
38	+++	AAAAGAGGAGATTC
39	+++	ATGTCGTCATGTGT
40	+++	AATGGCATGCTGCG
41	+++	AGAGGCAGTATCAA
43	+++	GTTTGGGTCCGGGC
72	+++	GGCATTTATGGGAA

**Table 3:** Nucleotide combinations that correlate with lack of reporter gene activation

Size	Sequence combination	Sensitivity	Specificity
12	R_5_-G_9_	6/6 (100%)	6/7 (86%)
12	K_6_-T_9_	8/9 (89%)	8/9 (89%)
20	W_8_-G_17_	5/8 (62%)	5/5 (100%)
20	A_11_-T_17_	5/5 (100%)	5/6 (83%)

IUPAC symbols: R = A or G; K = A or C; W = A or T.
