# Supplementary figures and images for: Flexible promoter architecture requirements for coactivator recruitment
Source: BMC Mol Biol. 2006 Apr 28;7:16. doi: 10.1186/1471-2199-7-16 (PMC1488866; doi:10.1186/1471-2199-7-16)

## Supplementary Figure 1

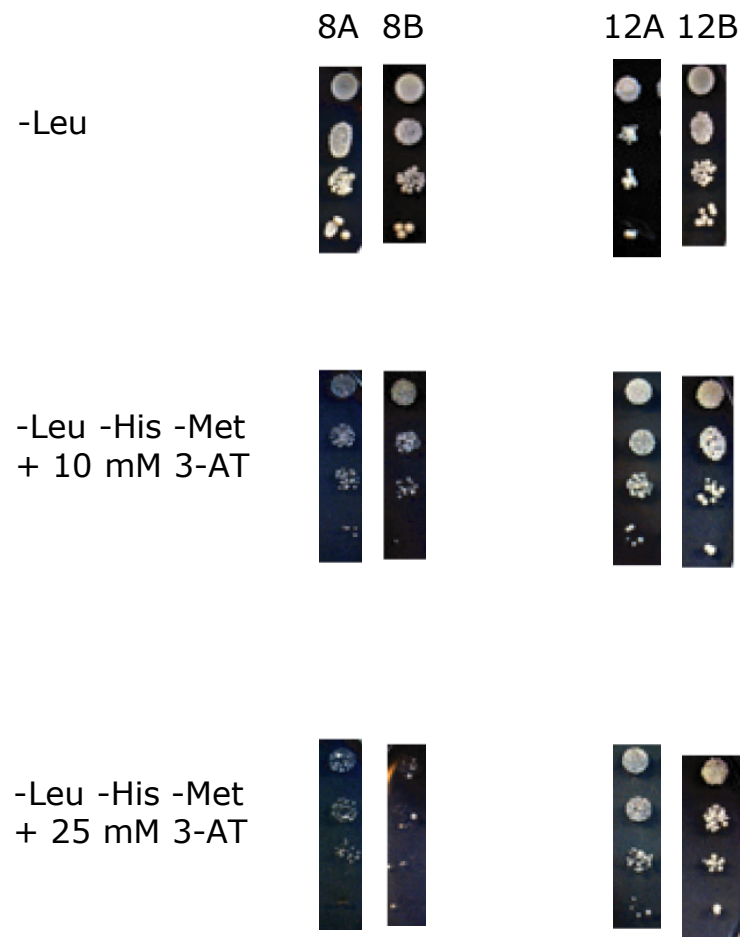

Supplement: Additional File 1 — Reproducibility of growth assays. Two different sequence contexts were tested between Cbf1 and Met31/32 binding sites with a center-to-center distance of 20 bp. The sequences of these clones were confirmed by isolating plasmids and sequencing. Clones 8A and 8B had the sequence TCACGTGTTTACAAACTAGGGGCCACA; clones 12A and 12B had the sequence TCACGTGGGCATTTATGGGAAGCCACA. These plasmids were transformed independently into yeast strains. Serial dilutions of separate isolates were plated on the indicated growth media. [file 1471-2199-7-16-S1.pdf]
